# Willingness to Pay for the COVID-19 Vaccine and Its Correlates in Bangladesh: Cross-Sectional Study

**DOI:** 10.2196/69827

**Published:** 2025-08-15

**Authors:** Mohammad Bellal Hossain, Md Zakiul Alam, Md Syful Islam, Shafayat Sultan, Md Mahir Faysal, Sharmin Rima, Md Anwer Hossain, Abdullah Al Mamun, Abdullah-Al- Mamun

**Affiliations:** 1 Department of Population Sciences, University of Dhaka, Third Floor, Arts Faculty Building, Dhaka, 1000, Bangladesh, 880 01766517002; 2 Laboratory of Fertility and Wellbeing, Max Planck Institute for Demographic Research, Rostock, Germany; 3 Department of Social Relations, East West University, Dhaka, Bangladesh; 4 Department of Japanese Studies, University of Dhaka, Dhaka, Bangladesh

**Keywords:** Bangladesh, willingness to pay, vaccines, COVID-19, infectious diseases, public health, public safety, cross-sectional study, financial

## Abstract

**Background:**

The Government of Bangladesh offers COVID-19 vaccines at no cost; however, sustaining this free vaccination program for a large population poses significant challenges. Thus, assessing the willingness to pay (WTP) for the COVID-19 vaccine is essential for understanding potential pricing strategies, subsidy requirements, and vaccine demand.

**Objective:**

This study aimed to assess the prevalence of WTP for the COVID-19 vaccine and its correlates.

**Methods:**

A cross-sectional design was used to collect data from 1497 respondents through web-based platform and face-to-face interviews. Multivariable logistic regression was used to analyze the correlates of the WTP.

**Results:**

The results showed that 772 of 1497 (51.6%) participants were willing to pay for the COVID-19 vaccine, with a median of 300 BDT (IQR 150-500 BDT; a currency exchange rate of 1 BDT=US $0.008 is applicable). The WTP was significantly higher among individuals with a graduate degree (adjusted odds ratio [aOR] 1.98, 95% CI 1.14-3.45) or master’s and MPhil or PhD-level education (aOR 1.93, 95% CI 1.07-3.48) and those with higher knowledge about the vaccine (aOR 1.09, 95% CI 1.02-1.15), positive behavioral practices (aOR 1.11, 95% CI 1.06-1.17), stronger subjective norms regarding COVID-19 vaccine (aOR 1.25, 95% CI 1.08-1.46), and higher anticipated regret of getting infected with COVID-19 (aOR 1.17, 95% CI 1.04-1.32). Conversely, WTP was lower among participants with negative attitudes toward vaccines (aOR 0.91, 95% CI 0.88‐0.95) and high perceived behavioral control regarding COVID-19 vaccination (aOR 0.86, 95% CI 0.76‐0.96; *P*=.006).

**Conclusions:**

With nearly half of the respondents unwilling to pay, this study highlights the need to improve vaccine-related knowledge and enhance income-based affordability to increase WTP. Health promotion efforts should focus on disseminating knowledge about vaccines and addressing negative perceptions. Additionally, a subsidized program for low-income groups can help mitigate financial barriers and promote equitable access to vaccines.

## Introduction

COVID-19 has caused tremendous health and socioeconomic challenges worldwide. As the treatment of COVID-19 is relatively expensive, preventing COVID-19 with a safe and effective vaccine is of utmost importance [1], which is considered one of the most successful public health interventions [2]. Even with safe and effective vaccines, the success of vaccination coverage programs relies on several other factors, such as willingness to vaccinate [3-6]. The intention to be vaccinated and the success of vaccination coverage programs have been influenced by the economic considerations of many people [7-9]. In this regard, willingness to pay (WTP) has emerged as a concept defined as the WTP any amount of money for a vaccine, health services, or technology [10-12].

To combat the vulnerability caused by COVID-19, the Government of Bangladesh (GoB) launched its largest vaccination program, providing vaccines free of charge. However, sustaining this free vaccination program is challenging [13] for a resource-poor country with a large population, such as Bangladesh. On the other hand, from the demand-side perspective, individuals’ out-of-pocket health expenditures in Bangladesh are already 74%, and 18.7% of the total population lives below the poverty line [14,15], making full payment for vaccines impossible. Thus, assessing WTP can offer insights into public demand and guide GoBs’ future pricing and payment strategies [1,3].

Few studies in Bangladesh have measured WTP in non–COVID-19 situations [8,16,17], whereas there is considerable evidence assessing WTP in the COVID-19 context in various countries [7,11,18]. WTP, both in COVID-19 and non–COVID-19 contexts, is determined by socioeconomic and demographic factors [3,8,11,19,20], health-related factors, knowledge about the disease and vaccine [7], different constructs of the health belief model, and behavioral factors [12].

However, there is scant evidence regarding WTP for the COVID-19 vaccine in Bangladesh. Existing studies conducted in Bangladesh to assess the prevalence and median WTP toward the COVID-19 vaccine [21] and associated factors [22] lacked representativeness of the Bangladeshi population, as the studies were only web-based and may result in an overrepresentation of more urban, younger, or technology-savvy individuals, skewing the sample and making it unreflective of the entire population. Thus, this study aimed to assess the prevalence of WTP for the COVID-19 vaccine and its correlates.

## Methods

### Study Design and Data Collection

This study used a cross-sectional design. The calculated sample size was 1635 using the formula (*z*^2^*pq*/*e*^2^)*Deff*NR. The *z* score for a 95% CI was 1.96, and the prevalence of willingness to accept a COVID-19 vaccine in an earlier study was 32.5% (*p*) [23]. We used a margin of error, *e*=0.03, for the sampling variation design effect (Deff)=1.6 and a 10% nonresponse rate. The ratio for face-to-face and web-based platform surveys was 2:1, considering the country’s digital divide. The overall participation rate was 93.1% (face-to-face survey: 91.9% and web-based platform survey: 97.7%), and 112 respondents did not consent to participate in the study (web-based platform survey: n=11 and face-to-face survey: n=101). Finally, 26 respondents who were unaware of the COVID-19 vaccine were excluded from the sample. Thus, the final sample for this study was 1497 for analysis (web-based platform survey: n=475, 31.7% and face-to-face survey: n=1022, 68.3%). As the GoB intended to initiate a mass vaccination program from February 7, 2021, we fixed February 1, 2021, to February 7, 2021, for data collection time.

Data were collected using a web-based platform through face-to-face interviews through Google Forms, where the questionnaire was developed in Bengali. Google Forms collects web-based data by prioritizing user friendliness and accessible design. This study conducted a pretest to validate and further improve the survey questionnaire. During the pretest, we tested the technical functionality and usability of the questionnaire. This helped us to identify and address missing questions, response codes, chronology, navigation, and technical performance. Due to the government’s restrictions on movement, safety protocols, and public hesitancy to meet data collectors at their homes, we decided to collect web-based data first to quickly reach a large audience. The survey link was circulated among the research team members’ networks via email, Facebook, WhatsApp, and other platforms. The respondents were also asked to share the link with their networks to reach as many people as possible. We used a single-response-per-participant option for the web-based survey to prevent duplicate entries. The web-based questionnaire contained 12 sections, each displayed on a separate screen for clarity. Upon completion, the respondents received a confirmation message indicating the successful submission of their responses. Additionally, the questionnaire included the respondents’ options to request the study results. Data collection for this study was completed between February 1 and 7, 2021 (web-based data were collected on February 1 and 3, 2021). After 3 days, the data were checked for divisional and age- and sex-specific distributions. To ensure representativeness of the sample, face-to-face interviews were conducted to determine the population’s national representation in terms of age, sex, residence, division, and marital status. Data were collected from 2 randomly selected districts in each of 8 divisions. Four days were allocated to collecting data through face-to-face interviews, and the interviewers maintained proper health measures during the interviews.

For offline data collection, the selection criteria to participate in this study were at least 18 years and older of age and knew about the COVID-19 vaccine, with an additional criterion for the web-based survey respondents’ reading and writing ability.

### Measures

#### Outcome Variables

Two questions were used to assess respondents’ WTP. The first question was, “Would you like to pay for the COVID-19 vaccine?” with a binary response (yes or no). If the response to the first question was “yes,” then the second question was, “What is the maximum amount you are willing to pay for the COVID-19 vaccine?”.

#### Independent Variables

##### Selection Process of Independent Variables

The independent variables in this study were selected through an extensive review of the literature. A range of socioeconomic, demographic, health status, COVID-19 infection, knowledge about the COVID-19 vaccine and vaccination process, conspiracy theories, preventive behavioral practices, and health behavioral models were selected. In health-related research, the theory of planned behavior (TPB), the health belief model, and 5C psychological antecedents are frequently used to capture attitudinal and normative influences on health behavior; however, we selected only the TPB because of its better predictability in such instances [24].

##### Socioeconomic and Demographic Variables

Several socioeconomic and demographic variables such as age, sex, religion, marital status, place of residence, household income, and occupation were used as independent variables in this study.

##### Perceived Health Status and COVID-19 Infection

Respondents’ perceived health status was assessed using the question, “How would you like to rate your health?” The response options were categorized as 1=bad, 2=moderate, and 3=good. Respondents were asked 3 separate binary (yes or no) questions regarding their experience with infection to assess their COVID-19 infection status: “Have you ever been infected with COVID-19? Has any member of your family been infected with COVID-19? Has any friend or acquaintance of yours contracted COVID-19?”

##### Knowledge of the COVID-19 Vaccine

Knowledge of the COVID-19 vaccine was assessed using four 5-point Likert-type items. The scores range from 1 to 20, with higher scores indicating greater knowledge. Cronbach α was 0.643. The commonly accepted Cronbach α threshold for measuring the internal consistency of the scales was 0.70. However, previous literature suggests that acceptance of moderate internal consistency is acceptable when measuring perceptual or cognitive-related issues, such as knowledge or susceptibility, and Cronbach α values between 0.60 and 0.70 can still be considered sufficient for complex psychological and health-related measures [25].

##### Knowledge About the Vaccination Process

Six questions with binary (yes=1 and no=0) response options were used to measure knowledge of the COVID-19 vaccination process, with a total score ranging from 0 to 6. Cronbach α was 0.765, indicating good internal consistency among the 6 questions. The higher the score, the better the understanding of the vaccination process.

##### COVID-19 Vaccine Conspiracy

Nine 5-point Likert-type items were used to assess conspiracy beliefs related to the COVID-19 vaccine [23], yielding scores ranging from 9 to 45, where a higher score indicated a stronger belief in conspiracy theories regarding the COVID-19 vaccine. Reliability analysis of these 9 questions revealed good internal consistency (α=.716).

##### Preventive Behavioral Practices Related to COVID-19

Preventive behavioral practices related to COVID-19 were measured using three 4-point items, with a composite value ranging from 1 to 12. A higher score indicates better preventive practices against COVID-19, and the items demonstrated excellent internal reliability (α=.857).

##### Theory of Planned Behavior

The TPB comprises 4 domains: attitude toward the COVID-19 vaccine, subjective norms toward the COVID-19 vaccine, perceived behavioral control against COVID-19 vaccination, and anticipated regret regarding getting infected by COVID-19.

###### Attitude Toward COVID-19 Vaccine

A 6-item 5-point Likert-type scale was used to assess attitudes related to the COVID-19 vaccine, with scores ranging from 6 to 30. A higher score on this scale indicates a more negative attitude toward the COVID-19 vaccine. Reliability analysis of these 6 items revealed good internal consistency (α=.739).

###### Subjective Norms Toward the COVID-19 Vaccine

Subjective norm refers to an individual’s perception of social pressure to perform or not perform a behavior. The subjective norm regarding the COVID-19 vaccine was assessed using a single 5-point item: “I believe my family members will support me in getting vaccinated against COVID-19.”

###### Perceived Behavioral Control Against COVID-19 Vaccination

Perceived behavioral control is defined as an individual’s belief about the ease or difficulty of performing a particular behavior, which can reflect experience and anticipated obstacles. It was measured using a single 5-point item: “I can register for COVID-19 vaccination if I want.”

###### Anticipated Regret Regarding Getting Infected by COVID-19

The anticipated regret of not getting vaccinated was assessed using a single 5-point item: “If I do not get a COVID-19 vaccine and end up getting Coronavirus, I will regret not getting the vaccination.”

### Statistical Analysis

At the beginning of the data analysis, we used poststratification weighting techniques to reduce biases stemming from sampling, thereby making our study representative of the national population of Bangladesh. We used a weight-adjustment technique for the age variable using the following formula:


$$\omega_{i}=\frac{p_{i}}{s_{i}}$$

where $\omega_{i}$ is the weight-adjusted factor, $p_{i}$ is the relative proportion of population characteristics, and $s_{i}$ is the proportion of sample characteristics. For example, the sample proportion for individuals aged 18‐24 years was 0.289, whereas the national population proportion, according to Bangladesh’s 2022 Population and Housing Census, was 0.201. Thus, using the formula, we calculated a weight of 1.43 for the age group 18‐24 years. Similarly, we calculated the weight of each age group to ensure that our study population was representative of the national population in terms of age distribution. The weighted samples were used for further analysis.

We first used univariate descriptive statistics (percentage, mean, and SD) for all variables to obtain the background characteristics of the participants and the prevalence of WTP for the COVID-19 vaccine. At the bivariate level, we obtained differentials in WTP for the COVID-19 vaccine using a chi-square test and point biserial correlation. Statistically significant variables (*P*≤.05) at the bivariate level were entered into a hierarchical logistic regression model to assess the correlates of WTP for the COVID-19 vaccine. We used only descriptive analysis (mean and median) for WTP the highest amount of money. Data were analyzed using SPSS (version 26; IBM Corp).

### Ethical Considerations

Ethics approval was obtained from the Bangladesh Medical Research Council (registration 39131012021). Voluntary participation was encouraged, and no incentives were provided to the participants. First, the aims, objectives, potential scopes, and implications of the study’s findings were communicated to the participants, and they then provided written consent. The respondents then participated in the study. Privacy and confidentiality were maintained throughout the study by ensuring that all participant data were anonymized and securely stored. Only authorized research personnel had access to the data, and the participants’ identities were kept confidential.

## Results

### Background Characteristics of the Participants

The background characteristics of the respondents are presented in Table 1, which shows that 340 of 1497 (22.7%) respondents in this study were between 31 and 39 years. Of the 1497 participants, 808 (54%) were male. In terms of religion, 1297 (86.7%) participants were Muslim, and 1061 (71%) participants were married. In total, 1129 (75.3%) had at least a secondary education and higher education. A total of 979 (66.4%) were from rural areas, and the highest percentage of respondents (n=474, 31.7%) were from the Dhaka division (Table 1). In total, 395 (26.4%) were homemakers. The mean number of household members was 4.95, and they had a mean collective monthly family income of 38,500 BDT (a currency exchange rate of 1 BDT=US $0.008 is applicable). Most of the participants (n=1074, 71.8%) identified themselves as having perfect health status (Table 1).

**Table 1.** Unweighted and weighted background characteristics of the respondents (N=1497).

Variables		Unweighted study sample	Weighted study sample
Age (years), n (%)			
	18‐24	432 (28.9)	300 (20.1)
	25‐30	362 (24.2)	295 (19.7)
	31‐39	254 (17.0)	340 (22.7)
	40‐49	236 (15.8)	276 (18.5)
	50+	213 (14.2)	286 (19.1)
Sex, n (%)			
	Female	692 (46.2)	689 (46.0)
	Male	805 (53.8)	808 (54.0)
Religion, n (%)			
	Muslim	1301 (86.9)	1297 (86.7)
	Others	196 (13.1)	200 (13.3)
Marital status, n (%)			
	Unmarried	575 (38.4)	436 (29.1)
	Married	922 (61.6)	1061 (70.9)
Education, n (%)			
	No education	129 (8.6)	158 (10.6)
	Primary	179 (12.0)	210 (14.1)
	Secondary and higher secondary	448 (29.9)	440 (29.4)
	Graduate	400 (26.7)	333 (22.2)
	Master’s and MPhil or PhD	341 (22.8)	356 (23.7)
Place of residence, n (%)			
	Rural	963 (64.3)	979 (66.4)
	Urban (other than city corporation)	179 (12.0)	171 (11.4)
	City corporation	355 (23.7)	347 (23.2)
Administrative division, n (%)			
	Barisal	114 (7.6)	118 (7.9)
	Chattogram	253 (16.9)	267 (17.8)
	Dhaka	478 (31.9)	474 (31.7)
	Khulna	137 (9.2)	132 (8.8)
	Mymensingh	108 (7.2)	106 (7.1)
	Rajshahi	180 (12.0)	179 (12.0)
	Rangpur	114 (7.6)	108 (7.2)
	Sylhet	113 (7.5)	113 (7.5)
Occupation, n (%)			
	Government, private, and NGO^a^ sector job	202 (13.5)	217 (14.5)
	Professionals	277 (18.5)	311 (20.8)
	Homemakers	348 (23.2)	395 (26.4)
	Students and unemployed	473 (31.6)	352 (23.5)
	Agriculture and day laborer	102 (6.81)	121 (8.1)
	Others	95 (6.34)	101 (6.7)
Collective monthly family income (BDT)^b^, n (%)			
	Less than 10,000	261 (17.4)	267 (17.9)
	10,000‐20,000	394 (26.3)	390 (26.1)
	20,000‐30,000	271 (18.1)	265 (17.7)
	30,000‐40,000	161 (10.8)	158 (10.6)
	40,000 and above	410 (27.4)	417 (27.8)
Perceived health status, n (%)			
	Bad or very bad	51 (3.4)	60 (4.0)
	Moderate	333 (22.2)	363 (24.3)
	Good or very good	1113 (74.3)	1074 (71.8)
Was infected with the coronavirus, n (%)			
	Yes	86 (5.7)	85 (5.7)
	No	1411 (94.3)	1412 (94.3)
Respondent’s family members were infected with the coronavirus, n (%)			
	Yes	152 (10.2)	148 (9.9)
	No	1345 (89.8)	1349 (90.1)
Respondent’s friends got infected with the coronavirus, n (%)			
	Yes	600 (40.1)	592 (39.6)
	No	897 (59.9)	905 (60.4)
Household size, mean (SD)		4.95 (1.98)	4.95 (2.05)

a NGO: nongovernmental organization.

b A currency exchange rate of 1 BDT=US $0.008 is applicable.

### Prevalence of WTP for the COVID-19 Vaccine and Its Differentials

The prevalence of WTP for the COVID-19 vaccine was 772 (51.6%) participants. However, 725 (48.4%) participants refused to pay any money for the COVID-19 vaccine.

Table 2 shows that WTP varied significantly (*P*≤.05) by religion, marital status, education, place of residence, administrative division, occupation, household income, and coronavirus infection status of respondents, family, and friends. For instance, respondents who identified as others than Muslim, unmarried, had master's and MPhil or PhD-level education, resided in an urban area, were from the Sylhet division, employed in a professional job, and had an income of 40,000 BDT and above had higher WTP at the bivariate level analysis (Table 2).

**Table 2.** Differentials of willingness to pay for COVID-19 vaccine among the study population using the chi-square test.

Variables		Willingness to pay		*P* value
		No, n (%)	Yes, n (%)	
Total		725 (48.4)	772 (51.6)
Age (years)				.16
	18‐24	140 (46.7)	160 (53.3)	
	25‐30	140 (47.5)	155 (52.5)	
	31‐39	154 (45.3)	186 (54.7)	
	40‐49	133 (48.2)	143 (51.8)	
	50+	157 (54.9)	129 (45.1)	
Sex				.72
	Female	330 (47.9)	359 (52.1)	
	Male	394 (48.8)	414 (51.2)	
Religion				<.001
	Muslim	653 (50.3)	644 (49.7)	
	Others	72 (36.0)	128 (64.0)	
Marital status				.01
	Unmarried	188 (43.2)	247 (56.8)	
	Married	537 (50.6)	525 (49.4)	
Education				<.001
	No education	111 (69.8)	48 (30.2)	
	Primary	132 (62.9)	78 (37.1)	
	Secondary and higher secondary	227 (51.6)	213 (48.4)	
	Graduate	129 (38.7)	204 (61.3)	
	Master’s and MPhil or PhD	126 (35.5)	229 (64.5)	
Place of residence				<.001
	Rural	526 (53.7)	453 (46.3)	
	Urban (other than city corporation)	61 (35.7)	110 (64.3)	
	City corporation	138 (39.8)	209 (60.2)	
Administrative division				.001
	Barisal	73 (61.9)	45 (38.1)	
	Chattogram	123 (46.1)	144 (53.9)	
	Dhaka	206 (43.5)	268 (56.5)	
	Khulna	70 (53.0)	62 (47.0)	
	Mymensingh	64 (60.4)	42 (39.6)	
	Rajshahi	87 (48.9)	92 (51.1)	
	Rangpur	55 (50.9)	53 (49.1)	
	Sylhet	47 (41.6)	66 (58.4)	
Occupation				<.001
	Government, private, and NGO^a^ sector job	88 (40.4)	129 (59.6)	
	Professionals	123 (39.5)	188 (60.5)	
	Homemakers	221 (55.9)	174 (44.1)	
	Students and unemployed	157 (44.7)	195 (55.3)	
	Agriculture and day laborer	77 (63.6)	44 (36.4)	
	Others	59 (58.4)	42 (41.6)	
Collective monthly family income (BDT)^b^				<.001
	Less than 10,000	156 (58.4)	111 (41.6)	
	10,000‐20,000	232 (59.5)	158 (40.5)	
	20,000‐30,000	127 (47.9)	138 (52.1)	
	30,000‐40,000	59 (37.3)	99 (62.7)	
	40,000 and above	151 (36.2)	266 (63.8)	
Perceived health status				.05
	Bad or very bad	30 (50.0)	30 (50.0)	
	Moderate	195 (53.7)	168 (46.3)	
	Good or very good	499 (46.5)	575 (53.5)	
Was infected with the coronavirus				.007
	Yes	29 (34.1)	56 (65.9)	
	No	696 (49.3)	716 (50.7)	
Respondent’s family members were infected with the coronavirus				.005
	Yes	55 (34.2)	93 (62.8)	
	No	669 (49.6)	680 (50.4)	
Respondent’s friends got infected with the coronavirus				<.001
	Yes	233 (39.4)	359 (60.6)	
	No	492 (54.4)	413 (45.6)	

a NGO: nongovernmental organization.

b A currency exchange rate of 1 BDT=US $0.008 is applicable.

Table 3 presents the point biserial correlation among WTP and independent variables. Knowledge about the COVID-19 vaccine and vaccination process, preventive behavioral practices related to COVID-19, subjective norm toward the COVID-19 vaccine, and anticipated regret regarding getting infected by COVID-19 had a statistically significant positive correlation with WTP. On the other hand, COVID-19 conspiracy beliefs, attitude toward the COVID-19 vaccine, and perceived behavioral control against COVID-19 vaccination were negatively correlated with WTP.

**Table 3.** Point biserial correlation between willingness to pay and selected independent variables.

Variables	Correlation coefficient (*r*)	*P* value
Household size	−0.038	.15
Knowledge about the COVID-19 vaccine	0.082	.001
Knowledge about the vaccination process	0.203	<.001
COVID-19 vaccine conspiracy	−0.165	<.001
Behavioral practice related to COVID-19	0.255	<.001
Attitude toward the COVID-19 vaccine	−0.315	<.001
Perceived behavioral control against COVID-19 vaccination	−0.163	<.001
Anticipated regret regarding getting infected by COVID-19	0.190	<.001
Subjective norms toward the COVID-19 vaccine	0.221	<.001

### Correlates of WTP for COVID-19 Vaccine

The significant variables at the bivariate level were entered into a hierarchical logistic regression model to assess the correlates of WTP for the COVID-19 vaccine. Three models were developed for this study. The first model was constructed using the socioeconomic and demographic variables. The second model was built using the variables from the first model along with knowledge of the COVID-19 vaccine and vaccination process, COVID-19 vaccine conspiracy theories, behavioral practices related to COVID-19, and health-related variables. Furthermore, the final model included all variables from the second model as well as components of the TPB. The final model showed that education, administrative division of Bangladesh, knowledge about the COVID-19 vaccine, behavioral practice related to COVID-19, attitude toward the COVID-19 vaccine, subjective norm toward COVID-19 vaccine, perceived behavioral control against COVID-19 vaccination, and anticipated regret regarding COVID-19 infection were statistically significant (*P≤* .05) predictors of WTP (Table 4).

**Table 4.** Correlates of willingness to pay for COVID-19 vaccine in Bangladesh using multivariable logistic regression (N=1497).

Variables	Model 1^a^, aOR^b^ (95% CI)	Model 2^c^, aOR (95% CI)	Model 3^d^, aOR (95% CI)
Religion: other (Muslim as RC^e^)	2.00 (1.43-2.81)^f^	1.75 (1.23-2.49)^g^	1.40 (0.97-2.01)^h^
Marital status: unmarried (married as RC)	1.08 (0.76-1.54)	1.09 (0.76-1.57)	1.23 (0.84-1.80)
Education (no education as RC)			
Primary	1.40 (0.89-2.22)	1.10 (0.68-1.78)	0.99 (0.60-1.62)
Secondary and higher secondary	2.09 (1.36-3.19)^g^	1.43 (0.92-2.24)	1.28 (0.80-2.04)
Graduate	3.36 (2.04-5.53)^f^	2.09 (1.23-3.55)^g^	1.98 (1.14-3.45)^h^
Master’s and MPhil or PhD	3.18 (1.87-5.41)^f^	1.82 (1.03-3.22)^h^	1.93 (1.07-3.48)^h^
Place of residence (rural as RC)			
Urban (other than city corporation)	1.40 (0.95-2.05)	1.11 (0.75-1.67)	1.1 (0.73-1.69)
City corporation	1.02 (0.74-1.42)	0.96 (0.67-1.36)	1.17 (0.81-1.69)
Administrative division of Bangladesh (Barisal as RC)			
Chattogram	1.80 (1.13-2.87)^h^	2.34 (1.43-3.84)^g^	2.27 (1.61-4.68)^f^
Dhaka	1.75 (1.13-2.72)^h^	1.22 (1.40-3.54)^g^	2.72 (1.66-4.45)^f^
Khulna	1.47 (0.86-2.51)	2.23 (1.27-3.92)^g^	3.37 (1.84-6.17)^f^
Mymensingh	0.96 (0.54-1.67)	1.01 (0.55-1.83)	1.13 (0.59-2.16)
Rajshahi	1.90 (1.14-3.15)^h^	2.54 (1.50-4.31)^g^	3.36 (1.19-5.91)^f^
Rangpur	1.49 (0.84-2.65)	2.11 (1.16-3.84)^h^	3.22 (1.70-6.09)^f^
Sylhet	2.80 (1.60-4.89)^g^	3.46 (1.93-6.21)^f^	4.70 (2.53-8.74)^f^
Occupation (government, private, and NGO^i^ sector job as RC)			
Professionals	1.15 (0.67-1.97)	1.04 (0.59-1.83)	1.18 (0.65-2.12)
Homemakers	1.56 (0.95-2.55)	1.40 (0.84-2.35)	1.50 (0.88-2.58)
Students and unemployed	1.58 (0.98-2.55)	1.70 (1.03-2.81)^h^	1.93 (1.14-3.25)^h^
Agriculture and day labor	1.17 (0.69-2.01)	1.21 (0.69-2.12)	1.34 (0.75-2.40)
Others	1.27 (0.71-2.28)	1.48 (0.80-2.72)	1.51 (0.80-2.85)
Income (BDT)^j^ (less than 10,000 as RC)			
10,000‐20,000	0.91 (0.65-1.27)	0.87 (0.61-1.22)	0.98 (0.68-1.40)
20,000‐30,000	1.16 (0.80-1.68)	1.11 (0.76-1.64)	1.25 (0.84-1.87)
30,000‐40,000	1.67 (1.07-2.59)^h^	1.56 (0.99-2.47)	1.48 (0.92-2.37)
40,000 and above	1.59 (1.07-2.37)*^h^	1.33 (0.88-2.02)	1.34 (0.87-2.06)
Knowledge about the COVID-19 vaccine	—^k^	1.13 (1.07-1.19)^f^	1.09 (1.02-1.15)^g^
Knowledge about the vaccination process	—	1.11 (1.04-1.19)^g^	1.06 (0.99-1.15)
Behavioral practice related to COVID-19	—	1.15 (1.09-1.20)^f^	1.11 (1.06-1.17)^f^
COVID-19 vaccine conspiracy	—	0.94 (0.91-0.97)^f^	1.01 (0.97-1.05)
Respondent got infected with coronavirus: yes (no as RC)	—	1.11 (0.66-1.87)	1.23 (0.71-2.12)
Respondent’s family member got infected with coronavirus: yes (no as RC)	—	1.09 (0.73-1.65)	1.08 (0.70-1.66)
Respondents’ friends or peers got infected with coronavirus: yes (no as RC)	—	1.15 (0.87-1.51)	1.16 (0.87-1.55)
Attitude toward the COVID-19 vaccine	—	—	0.91 (0.88-0.95)^f^
Subjective norms toward the COVID-19 vaccine	—	—	1.3 (1.12-1.51)^g^
Perceived behavioral control against COVID-19 vaccination	—	—	0.84 (0.75-0.94)^g^
Anticipated regret regarding getting infected by COVID-19	—	—	1.18 (1.06-1.32)^g^

a Constant=0.154; *P*<.001; −2 log likelihood=1924; Cox and Snell *R*2=0.095; Nagelkerke *R*2=0.127.

b aOR: adjusted odds ratio.

c Constant=0.028; *P*<.001; −2 log likelihood=1826; Cox and Snell *R*2=0.152; Nagelkerke *R*2=0.203.

d Constant=0.022; *P*<.001; −2 log likelihood=1730; Cox and Snell *R*2=0.206; Nagelkerke *R*2=0.274.

e RC: reference category.

f *P*<.001.

g *P*<.01.

h *P*<.05.

i NGO: nongovernmental organization.

j A currency exchange rate of 1 BDT=US $0.008 is applicable.

k Not available.

According to the final model, respondents of other religions were 40% more likely to pay for the COVID-19 vaccine than Muslims (adjusted odds ratio [aOR 1.4, 95% CI 0.97‐2.01). Similarly, education was a statistically significant predictor of WTP, where respondents who had a graduate degree (aOR 1.98, 95% CI 1.14‐3.45) and master’s and MPhil or PhD degrees (aOR 1.93, 95% CI 1.07‐3.480) had a higher WTP for the vaccine compared to the respondents who had no education. Division was a significant predictor of WTP, which showed that respondents from Chattogram (aOR 2.27, 95% CI 1.61‐4.68), Dhaka (aOR 2.72, 95% CI 1.66‐4.45), Khulna (aOR 3.37, 95% CI 1.84‐6.17), Rajshahi (aOR 3.36, 95% CI 1.19‐5.91), Rangpur (aOR 3.22, 95% CI 1.70‐6.09), and Sylhet (aOR 4.70, 95% CI 2.53‐8.74) had a higher WTP compared to respondents from Barisal. The findings also showed that WTP increased with an increasing level of knowledge about the COVID-19 vaccine (aOR 1.09, 95% CI 1.02‐1.15), behavioral practices related to COVID-19 (aOR 1.11, 95% CI 1.06-1.17), higher subjective norm toward COVID-19 vaccine (aOR 1.25, 95% CI 1.08-1.46), and higher anticipated regret regarding being infected by COVID-19 (aOR 1.17, 95% CI 1.04-1.32). On the other hand, a more negative attitude toward the COVID-19 vaccine (aOR 0.91, 95% CI 0.88-0.95) was associated with decreased WTP.

Although the logistic regression models initially included significant variables at the bivariate level, some variables lost their significance in the multivariate context. These included marital status, place of residence, occupation, income, and COVID-19 infection–related health status of the respondent, family, and friends. Compared to rural areas of residence, both urban and city corporations remain insignificant across the model. The respondent’s family income, notably when it exceeded 10,000 BDT, was negligible in the adjusted models. Among the administrative divisions, the Mymensingh division showed insignificant effects across all models. The COVID-19 infection–related health status of the respondents, family, and friends showed an insignificant association with the outcome variable.

### WTP the Highest Amount of Money

The mean and median WTP the highest amount of money among the respondents were 780.39 BDT and 300 BDT (IQR 150-500 BDT), respectively, for the COVID-19 vaccine. The WTP ranged from 1 BDT to 30,000 BDT.

**Figure 1.**
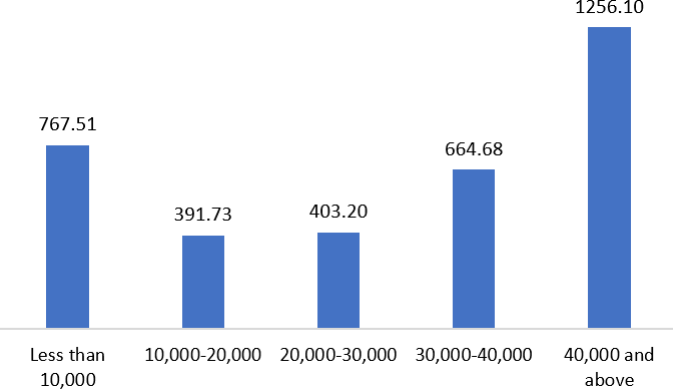
Respondents’ willingness to pay for the highest amount of money (average) by collective monthly family income category.

Figure 1 illustrates the mean WTP for collective monthly family income. There was an increasing trend in WTP, accompanied by rising collective monthly family income, except for the lowest income category (less than 10,000 BDT). Respondents from the 10,000‐20,000 BDT income group had a mean WTP of 391.73 BDT. On the other hand, the mean WTP of the group with an income of 40,000 BDT and above was 1256.10 BDT.

## Discussion

### Principal Findings and Comparison to Prior Work

A successful vaccination program depends not only on safe and effective vaccines but also on vaccine hesitancy and WTP. The objective of this study was to assess the prevalence of WTP for the COVID-19 vaccine and its correlates. The study found that 51.6% (n=772) of participants were willing to pay for the vaccine, with an average WTP of 780.49 BDT and a median of 300 BDT (IQR 150-500 BDT). Our findings reveal a lower prevalence and WTP compared to a previous study conducted in Bangladesh. A survey conducted earlier reported that a higher proportion of respondents (68.4%) were willing to pay for the vaccine, along with a higher median WTP of US $7.08 [21]. This difference may be attributed to differences in the sampling methodologies. An earlier study collected web-based data, which likely led to an overrepresentation of individuals who are more educated, financially better off, and digitally literate—characteristics associated with higher WTP. By contrast, our study used both web-based and offline (face-to-face) data collection methods, thereby capturing a more socioeconomically and educationally diverse sample, potentially leading to a more representative estimate of WTP in the general population.

Similarly, there were also relatively modest mean and median values among respondents from low- and middle-income countries who were willing to pay for the COVID-19 vaccine (mean WTP of US $5 in Ethiopia and US $15 for an 80% effective vaccination in Iran) [26]. Another study conducted in Indonesia revealed that the WTP for a COVID-19 vaccine booster dose in this low- and middle-income country remains relatively low, with only 66.2% of the respondents expressing WTP. Among them, the majority (63.5%) indicated a WTP within the range of US $6.71‐$33.57) [3]. As concerns about vaccine resistance and budgetary limitations create the expectation that the government will fully subsidize the vaccine, this expectation contributes to a lower willingness to accept and pay for the vaccine. However, the lower prevalence of WTP is a concern for attaining herd immunity, as it is estimated that 60% of the population must be immunized under a vaccination system to ensure the effectiveness of any vaccine [3].

Our findings suggest that religion has a significant impact on the WTP for the COVID-19 vaccine in Bangladesh. Muslims had a significantly lower WTP than other religions (Hindu, Christian, and Buddhist), and the existing literature supports these findings [3]. The Muslim community is known for its fatalistic view of health and its appreciation, acceptance, and patience regarding their current situation [27,28]. The majority of the population in Bangladesh is Muslim. Muslims may hold various religion-related beliefs, have lower perceptions and levels of knowledge about health and vaccines, and exhibit trust issues toward vaccines developed by non-Muslim countries. These factors can influence their decision to purchase a vaccine and contribute to a lower WTP for the COVID-19 vaccine [29-32].

Our study demonstrates that education has a significant predictive value for WTP for the COVID-19 vaccine [19,20]. In our study, respondents with a graduate and master's or MPhil or PhD-level of education had a higher WTP than those with no education. Educated individuals are more likely to be conscious of their health and more willing to prevent diseases through preventive care such as vaccination [26,33,34]. Education also influences knowledge of health and vaccines, and individuals with a low level of expertise are less likely to develop a WTP for vaccines [34,35]. Educated individuals are more likely to have higher incomes, which gives them greater purchasing power.

In our analysis, household income was found to have a significant positive association with WTP for the COVID-19 vaccine, particularly among higher-income groups. However, this association became statistically nonsignificant after adjusting for behavioral practices related to COVID-19 prevention. This suggests that behavioral practices may mediate the relationship between income and WTP. In our study sample, individuals with higher incomes were more likely to engage in protective behaviors against COVID-19. Consequently, the independent effect of income on WTP appeared to be mediated or absorbed by these behavioral practices, leading to the observed nonsignificant association after adjustment. However, income has proven to be one of the most influential factors in determining WTP for both COVID-19 and non–COVID-19 vaccines [8,18-20].

The study findings revealed a divisional disparity in WTP for the COVID-19 vaccine in Bangladesh. These differences may have occurred because of the socioeconomic and demographic differences among these divisions [36]. Barisal and Mymensingh are the 2 divisions with the highest poverty headcount ratios, whereas Sylhet, Dhaka, Khulna, and Chattogram have significantly lower poverty headcount ratios [37]. Our findings show that Sylhet has a 4-fold higher WTP, which may be attributed to economic and cultural factors. The household income and financial capacity of residents in Sylhet are high because of the strong remittance flow from the United Kingdom and the Middle East, which enhances their ability to pay for health services [38]. Additionally, the distinct cultural norms of the people of Sylhet may contribute to a higher WTP, as studies suggest that perceived norms and the personal environment are positively related to vaccination intention [38]. Households in Barisal and Mymensingh, with comparatively low incomes, may have a lower WTP for the COVID-19 vaccine. Our analysis reveals diverse WTP across divisions; however, a further qualitative study is essential to examine the behavioral and socioeconomic factors that underlie these disparities in WTP.

Apart from socioeconomic and demographic status, knowledge about the COVID-19 vaccine and preventive behavioral practices was associated with higher WTP, and the literature supports our study’s findings [7,39]. Increased knowledge about the COVID-19 vaccine indicates a more reliable understanding of its safety and effectiveness, which in turn improves trust in the vaccine; thus, WTP may increase. Again, people with higher preventive behavioral practices tend to be more educated and health-conscious, so they are expected to have a higher WTP toward any vaccine [40].

The study findings indicate that components of TPB are statistically significant predictors of WTP. The main argument of TPB is that behavioral intention is the most important determinant of health behavior, specifically in the case of WTP [41]. Our findings revealed that respondents with a more negative attitude toward vaccines also had a lower WTP. Attitude acts as a personal evaluation of a behavior; as a result, the intention to pay decreases with a more negative attitude [42]. Similarly, perceived behavioral control suggests that performing a health behavior is not solely within the respondent’s control; in this case, it is challenging to register. In our study, difficulty in web-based registration acts as a structural barrier that reduces WTP for the COVID-19 vaccine. On the other hand, our findings showed that subjective norms regarding COVID-19 vaccination and anticipated regret of contracting COVID-19 were positively associated with WTP for the vaccine. In our study, respondents whose family members approved of their decision to take the vaccine and highly regretted contracting COVID-19 had a higher WTP. This is also a part of subjective norms, as it provides permission from family members or friends for health behavior [41]. Thus, intention toward health behavior related to a disease or vaccine is driven by attitudes toward the disease or vaccine, subjective norms, perceived behavioral control, and other factors [41,43].

### Strengths and Limitations

This study aimed to assess the prevalence and correlates of WTP for the COVID-19 vaccine in Bangladesh, which can help the GoB and policy makers promote a successful vaccination program on a larger scale for the general population by addressing economic challenges. However, some limitations of this study should be considered prior to the present findings. Our study used a cross-sectional design, which is limited in its ability to generate causal inferences because of temporal issues. Nonprobability sampling was used to reach the study population. We collected self-reported data on health status and other sociodemographic variables, which may have been subject to recall bias. Furthermore, we collected data using both web-based and face-to-face methods. We acknowledge the potential for selection bias that may have resulted from these data-collection methods. Web-based respondents were more likely to be younger, urban, educated, and technology-savvy because of the digital nature of the data-collection platform, which may have ultimately resulted in an overrepresentation of these groups. To mitigate this, the majority of the data (n=1022, 68.3%) were collected through face-to-face interviews to ensure the representativeness of the sample and to determine the population’s national representation in terms of age, sex, residence, division, and marital status. However, it cannot be represented in terms of education, occupation, or income status. Finally, we acknowledge that WTP for a vaccine is context-dependent. Our study’s results may be influenced by the unique sociodemographic and cultural dynamics that emerged during the data collection.

### Conclusions

The study findings suggest that the government should introduce targeted educational campaigns aimed at specific demographics, such as religious communities, less educated groups, or those with lower incomes, to address their lower WTP for the COVID-19 vaccine. These campaigns need to be culturally appropriate to increase WTP. Health promotion materials and awareness campaigns as part of the behavior change communication program should be developed to increase knowledge about the COVID-19 vaccine. It will also increase preventive behavioral practices and reduce negative attitudes toward vaccines and vaccine-related conspiracies. Here, mass media can be an effective platform to circulate accurate messages of the COVID-19 vaccine, and community leaders, along with religious leaders, can also be incorporated to mitigate religion-related mistrust and misconceptions. Policy makers should reconsider the web-based registration procedure for vaccine uptake, as it poses a structural barrier to WTP for the COVID-19 vaccine. Based on our findings, an easy alternative system should be introduced for the mass population to achieve the sustainability of the vaccination program. The government may offer small incentives to those who choose vaccination, which will be particularly helpful for lower-income groups. We suggest that policy makers consider a subsidization program that considers socioeconomic stratification, with a focus on highlighting lower-income groups to mitigate the catastrophic income challenge associated with WTP for the COVID-19 vaccine. Otherwise, a reasonable price should be fixed so that the COVID-19 vaccine is affordable. This will help to achieve the highest vaccine coverage and run a successful vaccination program without economic hardship.

## Abbreviations

aOR: adjusted odds ratio
GoB: Government of Bangladesh
TPB: theory of planned behavior
WTP: willingness to pay
